# Greedy low-rank algorithm for spatial connectome regression

**DOI:** 10.1186/s13408-019-0077-0

**Published:** 2019-11-14

**Authors:** Patrick Kürschner, Sergey Dolgov, Kameron Decker Harris, Peter Benner

**Affiliations:** 1 Department of Electrical Engineering ESAT/STADIUS, KU Leuven, Leuven, Belgium; 2 Department of Mathematical Sciences, University of Bath, Bath, UK; 3 Paul G. Allen School of Computer Science & Engineering, Biology, University of Washington, Seattle, USA; 4 Computational Methods in Systems and Control Theory, Max Planck Institute for Dynamics of Complex Technical Systems, Magdeburg, Germany

**Keywords:** Matrix equations, Computational neuroscience, Low-rank approximation, Networks

## Abstract

Recovering brain connectivity from tract tracing data is an important computational problem in the neurosciences. Mesoscopic connectome reconstruction was previously formulated as a structured matrix regression problem (Harris et al. in Neural Information Processing Systems, 2016), but existing techniques do not scale to the whole-brain setting. The corresponding matrix equation is challenging to solve due to large scale, ill-conditioning, and a general form that lacks a convergent splitting. We propose a greedy low-rank algorithm for the connectome reconstruction problem in very high dimensions. The algorithm approximates the solution by a sequence of rank-one updates which exploit the sparse and positive definite problem structure. This algorithm was described previously (Kressner and Sirković in Numer Lin Alg Appl 22(3):564–583, 2015) but never implemented for this connectome problem, leading to a number of challenges. We have had to design judicious stopping criteria and employ efficient solvers for the three main sub-problems of the algorithm, including an efficient GPU implementation that alleviates the main bottleneck for large datasets. The performance of the method is evaluated on three examples: an artificial “toy” dataset and two whole-cortex instances using data from the Allen Mouse Brain Connectivity Atlas. We find that the method is significantly faster than previous methods and that moderate ranks offer a good approximation. This speedup allows for the estimation of increasingly large-scale connectomes across taxa as these data become available from tracing experiments. The data and code are available online.

## Introduction

Neuroscience and machine learning are now enjoying a shared moment of intense interest and exciting progress. Many computational neuroscientists find themselves inspired by unprecedented datasets to develop innovative methods of analysis. Exciting examples of such next-generation experimental methodology and datasets are large-scale recordings and precise manipulations of brain activity, genetic atlases, and neuronal network tracing efforts. Thus, techniques which summarize many experiments into an estimate of the overall brain network are increasingly important. Many believe that uncovering such network structures will help us unlock the principles underlying neural computation and brain disorders (Grillner et al. [17]). Initial versions of such connectomes (Knox et al. [25]) are already being integrated into large-scale modeling projects (Reimann et al. [40]). We present a method which allows us to perform these reconstructions faster, for larger datasets.

Structural connectivity refers to the synaptic connections formed between axons (outputs) and dendrites (inputs) of neurons, which allow them to communicate chemically and electrically. We represent such networks as a weighted, directed graph encoded by a nonnegative adjacency matrix *W*. The network of whole-brain connections or *connectome* is currently studied at a number of scales (Sporns [46], Kennedy et al. [24]): Microscopic connectivity catalogues individual neuron connections but currently is restricted to small volumes due to difficult tracing of convoluted geometries (Kasthuri et al. [23]). Macroscopic connectivity refers to connections between larger brain regions and is currently known for a number of model organisms (Buckner and Margulies [7]). Mesoscopic connectivity (Mitra [32]) lies between these two extremes and captures projection patterns of groups of hundreds to thousands of neurons among the 10^6^–10^10^ neurons in a typical mammalian brain.

Building on previous work (Harris et al. [19]; Knox et al. [25]), we present a scalable method to infer spatially-resolved mesoscopic connectome from tracing data. We apply our method to data from the Allen Mouse Brain Connectivity Atlas (Oh et al. [34]) to reconstruct mouse cortical connectivity. This resource is one of the most comprehensive publicly available datasets, but similar data are being collected for fly (Jenett et al. [22]), rat (Bota et al. [6]), and marmoset (Majka et al. [30]), among others. Our focus is on presenting and profiling an improved algorithm for connectome inference. By developing scalable methods as in this work, we hope to enable the reconstruction of high-resolution connectomes in these diverse organisms.

### Mathematical formulation of a spatial connectome regression problem

**Figure 1:**
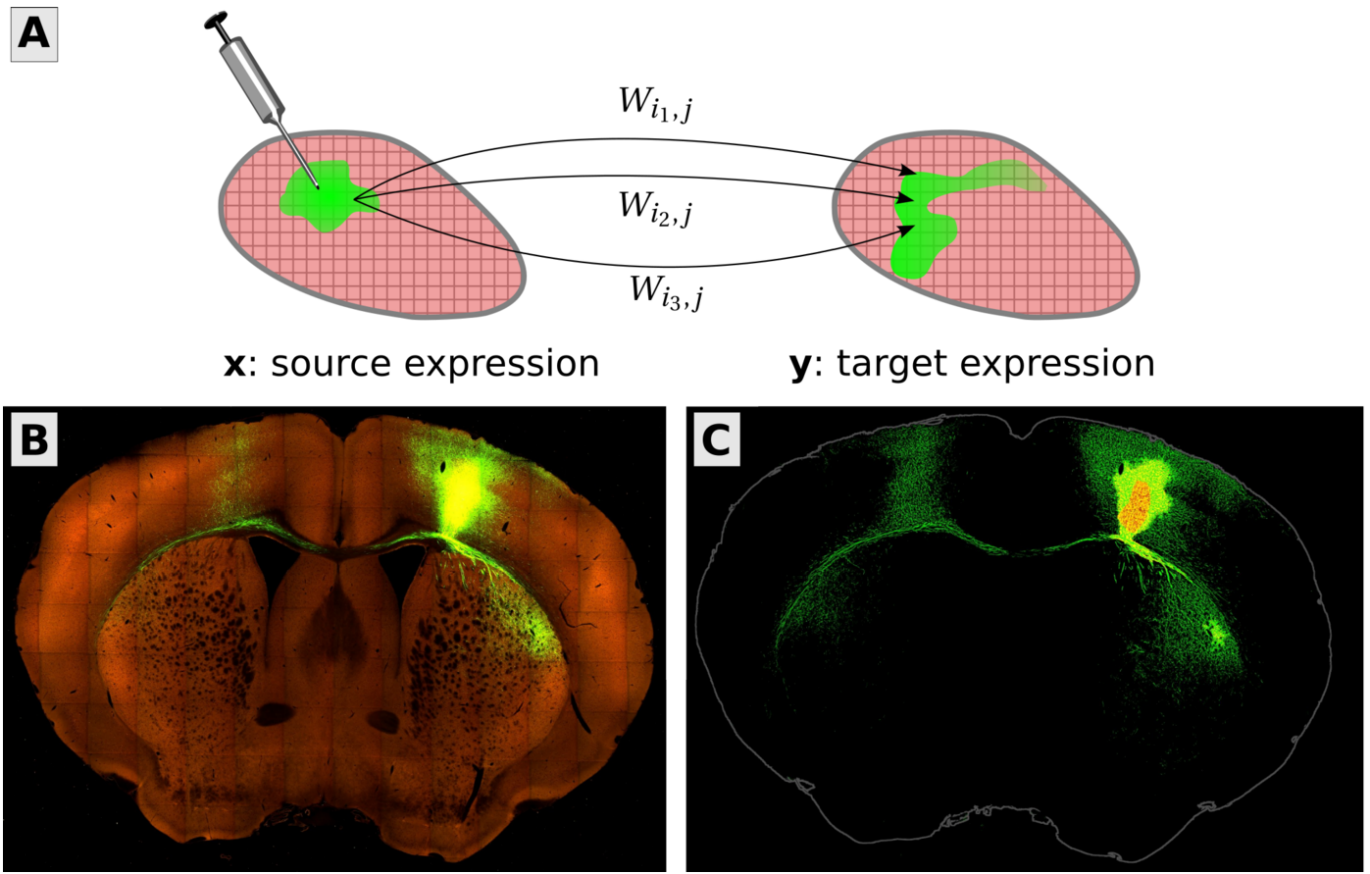
In this paper, we present an improved method for the mesoscale connectome inference problem. (A) The goal is to find a voxel-by-voxel matrix *W* so that the pattern of neural projections *y* arising from an injection *x* is reproduced by matrix-vector multiplication, $y \approx W x$. The vectors *x* and *y* contain the fraction of fluorescing pixels in each voxel from viral tracing experiments. (B) An example of the data, in this case a coronal slice from a tracing experiment delivered to primary motor cortex (MOp). Bright green areas are neural cells expressing the green fluorescent protein. (C) The raw data are preprocessed to separate the injection site (red/orange) from its projections (green). Fluorescence values in the injection site enter into the source vector *x*, whereas fluorescence everywhere else is stored in the entries of the target vector *y*. The *x* and *y* are discretized volume images of the brain reshaped into vector form. Entry $W_{ij}$ models the expected fluorescence at location *i* from one unit of source fluorescence at location *j*, a linear operator mapping from source images to target images. Image credit (B and C): Allen Institute for Brain Science

We focus on the mesoscale because it is naturally captured by viral tracing experiments (Fig. 1). In these experiments, a virus is injected into a specific location in the brain, where it loads the cells with proteins that can then be imaged, tracing out the projections of those neurons with cell bodies located in the injection site. The source and target signals, within and outside of the injection sites, are measured as the fraction of fluorescing pixels within cubic voxels. These form the data matrices $X\in \mathbb {R}^{{n_{\text{X}}}\times {n_{\text{inj}}}}$ and $Y\in \mathbb {R}^{{n_{\text{Y}}}\times {n_{\text{inj}}}}$, where parameters ${n_{\text{X}}}$ and ${n_{\text{Y}}}$ are the number of locations in the discretized source and target regions of the *d*-D brain, and ${n_{\text{inj}}}$ is the number of injections. In general, ${n_{\text{X}}}$ and ${n_{\text{Y}}}$ may be unequal, e.g. if injections were only delivered to the right hemisphere of the brain. Each experiment only traces out the projections from that particular injection site. By performing many such experiments, with multiple mice, and varying the injection sites to cover the brain, one can then “stitch” together a mesoscopic connectome for the average mouse. We refer the interested reader to (Oh et al. [34]) for more details of the experimental procedures.

We present a new low-rank approach to solving the smoothness-regularized optimization problem posed by Harris et al. [19]. Specifically, they considered solving the regularized least-squares problem

1 $$ W^{*} = \arg \min_{W \geq 0} \; \underbrace{\frac{1}{2} \bigl\Vert P_{ \varOmega } (WX-Y) \bigr\Vert _{F}^{2}}_{\text{loss}} + \underbrace{ \frac{ \lambda }{2} \bigl\Vert L_{y} W + W L_{x}^{\intercal }\bigr\Vert _{F}^{2}}_{ \text{regularization}}, $$

where the minimum is taken over nonnegative matrices. The operator $P_{\varOmega }$ defines an entry-wise product (Hadamard product) $P_{\varOmega } (M) = M \circ \varOmega $, for any matrix $M \in \mathbb{R} ^{{n_{\text{Y}}}\times {n_{\text{inj}}}}$, and *Ω* is a binary matrix, of the same size, which masks out the injection sites where the entries of *Y* are unknown.^1^ We take the smoothing matrices $L_{y}\in \mathbb{R}^{{n_{\text{Y}}}\times {n_{\text{Y}}}}$ and $L_{x}\in \mathbb{R}^{{n_{\text{X}}}\times {n_{\text{X}}}}$ to be discrete Laplace operator, i.e. the graph Laplacians of the voxel face adjacency graphs for discretized source and target regions. We choose a regularization parameter *λ̄* and set $\lambda = \bar{\lambda } \frac{{n_{\text{inj}}}}{{n_{\text{X}}}}$ to avoid dependence on ${n_{\text{X}}}, {n_{\text{Y}}}$ and ${n_{\text{inj}}}$, since the loss term is a sum over ${n_{\text{Y}}}\times {n_{\text{inj}}}$ entries and the regularization sums over ${n_{\text{Y}}}\times {n_{\text{X}}}$ many entries.

We now comment on the typical parameters for problem (1). The mouse brain gridded at 100 *μ*m resolution contains approximately ${n_{\text{X}}}, {n_{\text{Y}}}\in \mathcal{O}(10^{5})$ voxels in 3-D. On the other hand, the number of experiments ${n_{\text{inj}}}$ is less than $\mathcal{O}(10^{3})$. By projecting the 3-D cortical data into 2-D, as we do in this paper, we can reduce the size by an order of magnitude to ${n_{\text{X}}}, {n_{\text{Y}}}\in \mathcal{O}(10^{4})$, but focusing on the cortex reduces ${n_{\text{inj}}}$ to $\mathcal{O}(10^{2})$. Since ${n_{\text{inj}}}\ll {n_{\text{X}}}, {n_{\text{Y}}}$, a least-squares estimation of *W* (i.e. $\lambda = 0$) is highly underdetermined and will remain underdetermined unless orders of magnitude more tracing experiments are performed. Regularization is thus essential for filling the gaps in injection coverage. Furthermore, the vast size of the ${n_{\text{Y}}}\times {n_{\text{X}}}$ matrix *W* for whole-brain connectivities has motivated our search for scalable and fast low-rank methods.

### Previous methods of mesoscale connectome regression

Much of the work on mesoscale mouse connectomes leverages the data and processing pipelines of the Allen Mouse Brain Connectivity Atlas available at http://connectivity.brain-map.org (Lein et al. [28]; Oh et al. [34]). In the early examples of such work, Oh et al. [34] used viral tracing data to construct regional connectivity matrices. Nonnegative matrix regression was used to estimate the regional connectivity. First, the injection data were processed into a pair of matrices $X^{\text{Reg}}$ and $Y^{\text{Reg}}$ containing the regionalized injection volumes and projection volumes, respectively. The rows of these matrices are the regions and the columns index injection experiments. Oh et al. [34] then used nonnegative least squares to fit a region-by-region matrix $W^{\text{Reg}}$ such that $Y^{\text{Reg}} \approx W^{\text{Reg}} X^{\text{Reg}}$. Due to numerical ill-conditioning and a lack of data, some regions were excluded from the analysis. Similar techniques have been used to estimate regional connectomes in other animals. Ypma and Bullmore [49] took a different approach, using a likelihood-based Markov chain Monte Carlo method to infer regional connectivity and weight uncertainty from the Allen data.

Harris et al. [19] made a conceptual and methodological leap when they presented a method to use such data for spatially-explicit mesoscopic connectivity. The Allen Mouse Brain Atlas is essentially a coordinate mapping which discretizes the average mouse brain into cubic voxels, where each voxel is assigned to a given region in a hierarchy of brain regions. They used an assumption of spatial smoothness to formulate (1), where the specific smoothing term results in a high-dimensional thin plate spline fit (Wahba [48]). They then solved (1) using the generic quasi-Newton algorithm L-BFGS-B (Byrd et al. [8]). This technique was applied to the mouse visual areas but limited to small datasets since *W* was dense. Using a simple low-rank version based on projected gradient descent, Harris et al. [19] argued that such a method could scale to larger brain areas. However, the initial low-rank implementation turned out to be too slow to converge for large-scale applications. Times to convergence were not reported in the original paper, but the full-rank version typically took around a day, while the low-rank version needed multiple days to reach a minimum.^2^

Knox et al. [25] simplified the mathematical problem by assuming that the injections were delivered to just a single voxel at the injection center of mass. Using a kernel smoother led to a method which is explicitly low-rank, with smoothing performed only in the injection space (columns of *W*). This kernel method was applied to the whole mouse brain, yielding the first estimate of voxel–voxel whole-brain connectivity for this animal. However, these assumptions do not hold in reality: The injections affect a volume of the brain that encompasses much more than the center of mass.^3^ We also expect that the connectivity is also smooth across projection space (rows of *W*), since the incoming projections to a voxel are strongly correlated with those of nearby voxels. These inaccuracies mean that the kernel method is prone to artifacts, in particular ones arising from the injection site locations, since there is no ability for that method to translate the source of projections smoothly away from injection sites. It is thus imperative to develop an efficient method for the spline problem that works for large datasets.

### Continuous formulation motivates the need for sophisticated solvers

We will now describe, for the first time, the continuous mathematical properties of this problem, in order to illuminate why it is challenging to solve. Equation (1) can be seen as a discrete version of an underlying continuous problem (similar to Rudin et al. [42], among others), where we define the cost as

2 $$ \frac{1}{2} \sum_{i=1}^{{n_{\text{inj}}}}\int _{T \cap \varOmega _{i}} \biggl( \int _{S} \mathcal{W}(x,y) X_{i}(x) \,\mathrm{d}x - Y_{i}(y) \biggr)^{2} \,\mathrm{d}y + \frac{\lambda }{2} \int _{T} \int _{S} \bigl( \Delta \mathcal{W}(x,y) \bigr)^{2} \,\mathrm{d}x \,\mathrm{d}y . $$

The cost is minimized over $\mathcal{W}: T \times S \to \mathbb{R}$, the continuous connectome, in an appropriate Sobolev space (square-integrable derivatives up to fourth order on $T \times S$ is sufficiently regular). The function $\mathcal{W}$ may be seen as the kernel of an integral operator from *S* to *T*. These regions *S* and *T* are both compact subsets of $\mathbb{R}^{d}$ representing source and target regions of the brain. The mask region $\varOmega _{i} \subset T$ is the subset of the brain excluding the injection site. Finally, the discrete Laplacian terms *L* have been replaced by the continuous Laplacian operator Δ on $S \times T$. The parameter *λ* again sets the level of smoothing.^4^

For simplicity, consider $S = T =$ the whole brain, $\varOmega _{i} = T$ for all $i = 1, \ldots , {n_{\text{inj}}}$ and relax the constraint of nonnegativity on $\mathcal{W}$. Taking the first variational derivative of (2) and setting it to zero yields the Euler–Lagrange equations for this simplified problem:

3 $$\begin{aligned} 0 &= \lambda \Delta ^{2} \mathcal{W}(x,y) - \sum _{i=1}^{{n_{\text{inj}}}} X_{i}(x) Y_{i}(y) + \int _{S} \mathcal{W}\bigl(x',y\bigr) \Biggl( \sum_{i=1}^{{n_{\text{inj}}}} X _{i} \bigl(x'\bigr) X_{i}(x) \Biggr) \,\mathrm{d}x' \\ &= \lambda \Delta ^{2} \mathcal{W}(x,y) - g(x,y) + \int _{S} \mathcal{W}\bigl(x',y\bigr) f \bigl(x',x\bigr) \,\mathrm{d}x' , \end{aligned}$$

where for convenience we have defined the data covariance functions $f(x',x) = \sum_{i=1}^{{n_{\text{inj}}}} X_{i}(x') X_{i}(x) $ and $g(x,y) = \sum_{i=1}^{{n_{\text{inj}}}} X_{i}(x) Y_{i}(y) $, analogous to $X X^{T}$ and $YX^{T}$. The operator $\Delta ^{2}$ is the biharmonic operator or bi-Laplacian. Equation (3) is a fourth-order partial integro-differential equation in 2*d* dimensions.

Iterative solutions via gradient descent or quasi-Newton methods to biharmonic and similar equations can be slow to converge (Altas et al. [1]). It takes many iterations to propagate the highly local action of the biharmonic differential operator across global spatial scales due to the small stable step size (Rudin et al. [42]), whereas the integral part is inherently nonlocal. Very slow convergence is what we have found when applying methods like gradient descent to problem (1), also for low-rank versions. This includes quasi-Newton methods such as L-BFGS (Byrd et al. [8]). When we attempted to solve the whole-cortex top view and flatmap problems as in Sects. 3.2 and 3.3, the method had not converged (from a naive initialization) after a week of computation. These difficulties motivated the development of the method we present here.

### Outline of the paper

We present a greedy, low-rank algorithm tailored to the connectome inference problem. To leverage powerful linear methods, we consider solutions to the unconstrained problem

4 $$ W^{*} = \arg \min_{W} \frac{1}{2} \bigl\Vert P_{\varOmega } (WX-Y) \bigr\Vert _{F}^{2} + \frac{ \lambda }{2} \bigl\Vert L_{y} W + W L_{x}^{\intercal }\bigr\Vert _{F}^{2}, $$

where all of the matrices and parameters are as in (1). In practice, solutions to the linear problem (4) are often very close to those of the nonnegative problem (1), since the data matrices *X* and *Y* and the “true” *W* are nonnegative. Setting any negative entries in the computed solution $W^{*}$ to zero is adequate, or it can serve as an initial guess to an iterative solver for the slower nonnegative problem.

Equation (4) is another regularized least-squares problem. In Sect. 2.1, we show that taking the gradient and setting it equal to zero leads to a linear matrix equation in the unknown *W*. This takes the form of a generalized Sylvester equation with coefficient matrices formed from the data and Laplacian terms. The data matrices are, in fact, of low rank since ${n_{\text{inj}}}\ll {n_{\text{X}}}, {n_{\text{Y}}}$, and thus we can expect a low-rank approximation $W \approx UV^{\intercal }$ to the full solution to perform well (see Harris et al. [19], although we do not know how to justify this rigorously). We provide a brief survey of some low-rank methods for linear matrix equations in Sect. 2.2. We employ a greedy solver that finds rank-one components $u_{i} v_{i}^{\intercal }$ one at a time, explained in Sect. 2.3. After a new component is found, it is orthogonalized and a Galerkin refinement step is applied. This leads to Algorithm 1, our complete method.

**Algorithm 1:**
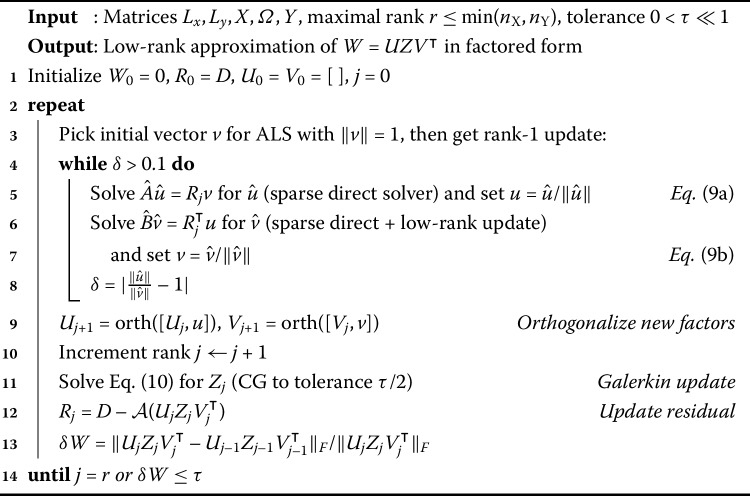
Greedy rank-1 method with Galerkin projection for (7)

We then test the method on a few connectome fitting problems. First, in Sect. 3.1, we test on a fake “toy” connectome, where we know the truth. This is a test problem consisting of a 1-D brain with smooth connectivity (Harris et al. [19]). We find that the output of our algorithm converges to the true solution as the rank increases and as the stopping tolerance decreases. Next, we present two benchmarks using real viral tracing data from the isocortices of mice, provided by the Allen Institute for Brain Science. In each case, we work with 2-D data in order to limit the problem size and because the cortex is a relatively flat, 2-D shape. It has also been argued that such a projection also denoises such data (Van Essen [47]; Gămănuţ et al. [14]). In Sect. 3.2, we work with data that are averaged directly over the superior-inferior axis to obtain a flattened cortex. We refer to this as the *top view* projection. In contrast, for Sect. 3.3, the data are flattened by averaging along curved streamlines of cortical depth. We call this the *flatmap* projection.

Finally, Sect. 4 discusses the limitations of our method and directions for future research. Our data and code are described in Sect. 5 and freely available for anyone who would like to reproduce the results.

## Greedy low-rank method

### Linear matrix equation for the unknown connectivity

We now derive the equivalent of the “normal equations” for our problem. Denote the objective function (4) as $J(W)$, with decomposition

$$J(W) = J_{\mathrm{loss}}(W) + J_{\mathrm{reg}}(W) = \frac{1}{2} \bigl\Vert P _{\varOmega } (WX-Y) \bigr\Vert _{F}^{2} + \frac{\lambda }{2} \bigl\Vert L_{y} W + W L_{x} ^{\intercal }\bigr\Vert _{F}^{2}. $$

Writing $J_{\mathrm{loss}}$ indexwise, we obtain (note that $\varOmega \circ \varOmega =\varOmega $)

$$J_{\mathrm{loss}} = \frac{1}{2}\sum_{i,\alpha =1}^{n, {n_{\text{inj}}}} \varOmega _{i,\alpha } \Biggl(\sum_{k=1}^{m} W_{i,k} X_{k,\alpha } - Y _{i,\alpha } \Biggr)^{2}. $$

The derivative reads

$$\begin{aligned} \frac{\partial J_{\mathrm{loss}}}{\partial W_{\hat{\imath },\hat{k}}} &= \sum_{i,\alpha =1}^{n,{n_{\text{inj}}}} \varOmega _{i,\alpha } \Biggl( \sum_{k=1} ^{m} W_{i,k} X_{k,\alpha } - Y_{i,\alpha } \Biggr) X_{\hat{k},\alpha } \delta _{i,\hat{\imath }} \\ &= \sum_{\alpha =1}^{{n_{\text{inj}}}} \varOmega _{\hat{\imath },\alpha } X_{ \hat{k},\alpha } \sum_{k=1}^{m} ( X_{k,\alpha } W_{ \hat{\imath },k} - X_{\hat{k},\alpha } \varOmega _{\hat{\imath },\alpha } Y_{\hat{\imath },\alpha } ) , \end{aligned}$$

or in vector form

$$ \frac{\partial J_{\mathrm{loss}}}{\partial \operatorname{vec}(W)} = \sum_{\alpha =1}^{{n_{\text{inj}}}} \bigl[ \bigl(X_{\alpha } X_{\alpha }^{\intercal }\bigr) \otimes \operatorname{diag}(\varOmega _{\alpha }) \bigr] \operatorname{vec}(W) - \operatorname{vec} \bigl((\varOmega \circ Y) X^{\intercal }\bigr), $$

where $X_{\alpha }$ is the *α*th column of *X* and likewise for *Ω*. Setting the derivative equal to zeros leads to the system of normal equations

5 $$ A \operatorname{vec}(W) = \operatorname{vec} \bigl((\varOmega \circ Y) X^{\intercal }\bigr) , $$

where $\operatorname{vec}(W)$ is the vector of all columns of *W* stacked on top of each other. This linear system features the following $({n_{\text{Y}}}{n_{\text{X}}}) \times ({n_{\text{Y}}}{n_{\text{X}}})$ matrix, consisting of ${n_{\text{inj}}}+3$ Kronecker products,

6 $$ A = \sum_{\alpha =1}^{{n_{\text{inj}}}}\bigl(X_{\alpha } X_{\alpha }^{\intercal }\bigr) \otimes \operatorname{diag}( \varOmega _{\alpha }) + \lambda \bigl(L_{x}^{2} \otimes I_{{n_{\text{Y}}}}+ 2 L_{x} \otimes L_{y} + I_{{n_{\text{X}}}}\otimes L_{y}^{2}\bigr) . $$

Note that without the observation mask, *Ω* is a matrix of all ones, and the first term compresses to $XX^{\intercal }\otimes I_{{n_{\text{Y}}}}$.

The linear system (5) can be recast as the linear matrix equation

7 $$\begin{aligned} \mathcal{A}(W) = D, \end{aligned}$$

with the operator $\mathcal{A}(W):=\lambda \mathcal{B}(W)+ \mathcal{C}(W) $, where

$$\begin{aligned}& \mathcal{B}(W):=WL^{2}_{x}+2L_{y}WL_{x}+L^{2}_{y}W, \\& \mathcal{C}(W):= \sum_{\alpha =1}^{{n_{\text{inj}}}}\operatorname{diag} (\varOmega _{\alpha }) WX_{\alpha } X_{\alpha }^{\intercal }, \quad \text{and}\quad D:=(\varOmega \circ Y) X^{\intercal }. \end{aligned}$$

The smoothing term $\mathcal{B}$ can be expressed as a squared standard Sylvester operator $\mathcal{B}(W)=\mathcal{L}(\mathcal{L}(W)) $, where $\mathcal{L}(W):=L_{y} W + W L_{x}$. The operator $\mathcal{L}$ is the graph Laplacian operator on the discretization of $T \times S$. Furthermore, the right hand side *D* is a matrix of rank ${n_{\text{inj}}}$, since it is an outer product of two rank ${n_{\text{inj}}}$ matrices.

### Numerical low-rank methods for linear matrix equations

Because of the potentially high dimensions ${n_{\text{X}}},{n_{\text{Y}}}$, directly solving the algebraic matrix equation (7) is numerically inefficient since the solution will be a dense ${n_{\text{Y}}}\times {n_{\text{X}}}$ matrix, making even storing it infeasible. However, the rank of the right hand side of (7) is at most ${n_{\text{inj}}}\ll {n_{\text{X}}},{n_{\text{Y}}}$. It is often observed and theoretically shown (Grasedyck [16]; Benner and Breiten [3]; Jarlebring et al. [21]) that the solutions of large matrix equations with low-rank right hand sides exhibit rapidly decaying singular values. Hence, the solution *W* is expected to have small numerical rank in the sense that few of its singular values are larger than machine precision or the experimental noise floor. Intuitively, since we also seek very smooth solutions, this also helps control the rank, since high frequency components tend to be associated with small singular values. This motivates us to approximate the solution of (7) by a low-rank approximation $W\approx UV^{\intercal }$ with $U\in \mathbb {R}^{{n_{\text{Y}}}\times r}$, $V\in \mathbb {R}^{{n_{\text{X}}}\times r}$ and $r\ll \min ({n_{\text{X}}},{n_{\text{Y}}})$. The low-rank factors are then typically computed by iterative methods which never form the approximation $UV^{\intercal }$ explicitly.

Several low-rank methods for computing $U,V$ have been proposed, starting from methods for standard Sylvester equations $AX + XB = D$ (e.g. Benner [2]; Benner et al. [4]; Benner and Saak [5]; Simoncini [44]) and more recently for general linear matrix equations like (7) (Damm [13]; Benner and Breiten [3]; Shank et al. [43]; Ringh et al. [41]; Jarlebring et al. [21]; Powell et al. [39]). However, these methods are specialized and require the problem to have particular structures or properties (e.g., $\mathcal {B}, \mathcal {C}$ have to form a convergent splitting of $\mathcal {A}$), which are not present in the problem at hand. The main structures present in (7) are positive definiteness and sparsity of $L_{x}, L_{y}$.

An approach that is applicable to the matrix equation (7) is a greedy method as proposed by Kressner and Sirković [26], which is based on successive rank-1 approximations of the error. Because this method is quite general, we tailored specific components of the algorithm to our problem. Three main challenges were overcome: First, we choose a simpler stopping criterion for the ALS routine. Second, specific solvers were chosen for the three main sub-problems of the algorithm, which maximizes its efficiency. Third, we developed a GPU implementation of the Galerkin refinement, to make this bottleneck step more efficient. We advocate this method in the rest of the paper.

### Description and application of the greedy low-rank solver

Here we briefly review the algorithm from (Kressner and Sirković [26]) and explain how it is specialized for our particular problem. Assume there is already an approximate solution $W_{j}\approx W^{*}$ of the linear matrix equation $\mathcal{A}(W) = D$, equation (7), with solution $W^{*}$. We will improve our solution by an update of rank one: $W_{j+1}=W_{j}+u_{j+1}v_{j+1}^{\intercal }$, where $u_{j+1}\in \mathbb{R}^{{n_{\text{Y}}}}$ and $v_{j+1}\in \mathbb{R}^{{n_{\text{X}}}}$. The update vectors $u_{j+1}$, $v_{j+1}$ are computed by minimizing an error functional that we will soon define. Since the operator $\mathcal{A}$ is positive definite, it induces the $\mathcal{A}$-inner product $\langle X,Y\rangle _{\mathcal{A}} = \operatorname{Tr}\! (Y^{\intercal } \mathcal{A}(X) )$ and the $\mathcal{A}$-norm $\|Y\|_{ \mathcal{A}}:=\sqrt{\langle Y,Y\rangle _{\mathcal{A}}}$. So we find $u_{j+1}$, $v_{j+1}$ by minimizing the squared error in the $\mathcal{A}$-norm:

$$\begin{aligned} (u_{j+1},v_{j+1} ) &= \arg \min_{u,v} \bigl\Vert W^{*} - W_{j} - uv ^{\intercal }\bigr\Vert _{\mathcal{A}}^{2} \\ &= \arg \min_{u,v} \operatorname{Tr}\! \bigl( \bigl(W^{*} - W_{j} - uv ^{\intercal }\bigr)^{\intercal }\mathcal{A}\bigl(W^{*} - W_{j} - uv^{\intercal }\bigr) \bigr) \\ &= \arg \min_{u,v} \operatorname{Tr}\! \bigl( \bigl(W^{*} - W_{j} - uv ^{\intercal }\bigr)^{\intercal }\bigl(D - \mathcal{A}(W_{j}) - \mathcal{A} \bigl(uv^{\intercal }\bigr)\bigr) \bigr). \end{aligned}$$

Discarding constant terms, noting that $\langle X, Y \rangle _{ \mathcal{A}}= \langle Y,X \rangle _{\mathcal{A}}$, and setting $R_{j}=D-\mathcal{A}(W_{j})$ leads to

8 $$\begin{aligned} (u_{j+1},v_{j+1} ) = \arg \min _{u,v} \bigl\langle uv^{\intercal },uv ^{\intercal }\bigr\rangle _{\mathcal{A}}-2\operatorname{Tr}\! \bigl(uv^{\intercal }R _{j} \bigr). \end{aligned}$$

Notice that the rank-1 decomposition $u v^{\intercal }$ is not unique, because we can rescale the factors by any nonzero scalar *c* such that $(uc)(v/c)^{\intercal }$ represents the same matrix. This reflects the fact that the optimization problem (8) is not convex. However, it is convex in each of the factors *u* and *v* separately.

We obtain the updates $u_{j+1}$, $v_{j+1}$ via an alternating linear (ALS) scheme (Ortega and Rheinboldt [35]). Although we only consider low-rank approximations of matrices here, ALS methods are also used for computing low-rank approximations of higher order tensors by means of polyadic decompositions (e.g. Harshman [20]; Sorber et al. [45]). First, a fixed *v* is used in (8) and a minimizing *u* is computed which is in the next stage kept fixed and (8) is solved for a minimizing *v*. For a fixed vector *v* with $\|v\|=1$ the minimizing problem is

$$\begin{aligned} \hat{u} &= \arg \min_{u} \bigl\langle uv^{\intercal },uv^{\intercal }\bigr\rangle _{\mathcal{A}}-2\operatorname{Tr}\! \bigl(uv^{\intercal }R_{j} \bigr) \\ \begin{aligned} &=\arg \min_{u} \Biggl(\lambda \bigl(\bigl(u^{\intercal }u\bigr)v^{\intercal }L_{x} ^{2}v+2\bigl(u^{\intercal }L_{y} u\bigr) \bigl(v^{\intercal }L_{x}v\bigr)+u^{\intercal }L_{y}^{2}u \bigr) \\ & \quad +\sum_{\alpha =1}^{{n_{\text{inj}}}}\bigl(u^{\intercal }\operatorname{diag}( \varOmega _{\alpha })u\bigr) \bigl(v^{\intercal }X_{\alpha } X_{\alpha }^{\intercal }v \bigr) \Biggr)-2u^{\intercal }R_{j}v \end{aligned} \end{aligned}$$

and, hence, *û* is obtained by solving the linear system of equations

9a $$\begin{aligned} \hat{A} \hat{u}=R_{j}v,\qquad \hat{A}:= \lambda \bigl(\bigl(v^{\intercal }L _{x}^{2}v \bigr)I+2L_{y} \bigl(v^{\intercal }L_{x}v \bigr)+L_{y}^{2} \bigr) + \sum _{\alpha =1}^{{n_{\text{inj}}}}\operatorname{diag}(\varOmega _{\alpha }) \bigl(v^{\intercal }X_{\alpha } X_{ \alpha }^{\intercal }v\bigr). \end{aligned}$$

The second half iteration starts from the fixed $u=\hat{u}/\|\hat{u} \|$ and tries to find a minimizing *v̂* by solving

9b $$\begin{aligned}& \begin{gathered} \hat{B} \hat{v}=R^{\intercal }_{j}u,\\ \hat{B}:=\lambda \bigl( L_{x} ^{2}+2L_{x} \bigl(u^{\intercal }L_{y}u\bigr)+\bigl(u^{\intercal }L_{y}^{2}u\bigr)I \bigr) + \sum _{\alpha =1}^{{n_{\text{inj}}}}\bigl(u^{\intercal }\operatorname{diag}(\varOmega _{\alpha })u\bigr) \bigl(X_{\alpha } X_{ \alpha }^{\intercal }\bigr) \end{gathered} \end{aligned}$$

which can be derived by similar steps. The linear systems (9a) and (9b) inherit the sparsity from $L_{x}$, $L_{y}$ and *Ω*. Therefore they can be solved by sparse direct or iterative methods. We use a sparse direct solver for (9a), as this was faster than the alternatives. The coefficient matrix *B̂* in (9b) is the sum of a sparse (Laplacian terms) matrix and a low-rank (rank ${n_{\text{inj}}}$ data terms) matrix. In this case, we solve (9b) using the Sherman–Morrison–Woodbury formula (Golub and Van Loan [15]) and a direct solver for the sparse inversion.

Both half steps form the ALS iteration which should be stopped when the iterates are close enough to a critical point, which might be difficult to check. Here we propose a simpler approach compared to the one in (Kressner and Sirković [26]). Since we rescale *u* and *v* such that $\|u\|_{2}= \|v\|_{2}=1$, the norm of the other factor is equal to the norm of the full matrix. In other words, $\|\hat{u}\|_{2} = \|\hat{u}v^{\intercal }\|_{2}$ after solving for *û*, and hence $\| \hat{u} \|_{2}$ should converge to the norm of the exact solution. This motivates a simple criterion: we stop the ALS when $(1-\delta ) \|\hat{u}\|_{2} \le \|\hat{v}\|_{2} \le (1+\delta ) \|\hat{u}\|_{2} $, where *û* and *v̂* are taken from two consecutive ALS steps, and $\delta <1$ is a small threshold. It turns out that a relatively crude tolerance of $\delta =0.1$, corresponding to 2–4 ALS iterations, is sufficient in practice for the overall convergence of the algorithm.

The second stage of the method is a non-greedy Galerkin refinement of the low-rank factors. Suppose a rank *j* approximation $W_{j}=\sum_{i=1}^{j} u_{i} v_{i}^{\intercal }$ of *W* has been already computed. Let $U \in \mathbb{R}^{{n_{\text{Y}}}\times j}$ and $V\in \mathbb{R}^{{n_{\text{X}}}\times j}$ have orthonormal columns, spanning the spaces $\mathrm{span}\lbrace u _{1},\ldots ,u_{j}\rbrace $ and $\mathrm{span}\lbrace v_{1},\ldots ,v _{j}\rbrace $, respectively. We compute a refined approximation $UZV^{\intercal }$ for $Z\in \mathbb{R}^{j\times j}$ by imposing the following condition onto the residual:

$$\begin{aligned} &\text{(Galerkin condition)}\\ &\quad \text{Find }Z\text{ so that} \quad \mathcal{A}\bigl(UZV ^{\intercal }\bigr) - D \quad \perp \quad \bigl\lbrace UZV^{\intercal }\in \mathbb{R} ^{{n_{\text{Y}}}\times {n_{\text{X}}}}, Z\in \mathbb{R}^{j\times j}\bigr\rbrace . \end{aligned}$$

This leads to the dense, square matrix equation in *Z* of dimension $j \leq r\ll {n_{\text{X}}},{n_{\text{Y}}}$:

10 $$\begin{aligned} &\lambda \bigl(Z\bigl(V^{\intercal }L^{2}_{x}V \bigr) + 2\bigl(U^{\intercal }L_{y}U\bigr)Z\bigl(V^{\intercal }L _{x} V\bigr)+\bigl(U^{\intercal }L^{2}_{y}U \bigr)Z \bigr) \\ &\quad {} +\sum_{\alpha =1}^{{n_{\text{inj}}}}\bigl(U ^{\intercal }\operatorname{diag}(\varOmega _{\alpha }) U\bigr)Z \bigl(V^{\intercal }X_{\alpha } X _{\alpha }^{\intercal }V \bigr) = U^{\intercal }D V . \end{aligned}$$

Equation (10) is a projected version of (7) and inherits its structure including the positive definiteness of the operator which acts on *Z*. Instead of using a direct method to solve (10) (as in Kressner and Sirković [26]), we employ an iterative method similar to Powell et al. [39]. Due to the positive definiteness, the obvious method of choice is a dense, matrix-valued conjugate gradient method (CG). Moreover, we reduce the number of iterations significantly by taking the solution *Z* from the previous greedy step as an initial guess. The improved solution $W_{j+1}=UZV^{\intercal }$ yields a new residual $R_{j+1}=D-\mathcal{A}(W_{j+1})$ onto which the ALS scheme is applied to obtain the next rank-1 updates. The complete procedure is illustrated in Algorithm 1.

This Galerkin refinement substantially improves the greedy approximation, leading to a faster convergence rate (Kressner and Sirković [26]). The ALS stage is primarily used to sketch the projection bases for the Galerkin solution, which justifies the limited number of ALS steps. Use of the Galerkin refinement in the low-rank decomposition literature can be traced back to the greedy approximation in the CP tensor format (Nouy [33]), as well as orthogonal matching pursuit approaches in sparse recovery and compressed sensing (Pati et al. [37]) and deflation strategies in low-rank matrix completion (Hardt and Wootters [18]).

## Performance of the greedy low-rank solver on three problems

There are three test problems to which we apply Algorithm 1: a toy problem with synthetic data (Sect. 3.1), the top view projected mouse connectivity data (Sect. 3.2), and the flatmap projected data (Sect. 3.3). These tests show that the method easily scales to whole-brain connectome reconstruction.

We investigate the computational complexity and convergence of the greedy algorithm. Since the matrices in (9a) are sparse, the ALS steps need $\mathcal{O}(nr^{2} {n_{\text{inj}}})$ operations in total for the final solution rank *r*, where $n = \max ({n_{\text{X}}},{n_{\text{Y}}})$. In turn, if the solution of (10) takes *γ* CG iterations, this step will have a cost of $\mathcal{O}(\gamma r^{3} {n_{\text{inj}}})$. Although *γ* can be kept at the same level for all *j*, it depends on the stopping tolerance *τ*, as does the rank *r*. We will therefore investigate the cost in terms of the total computation time and the corresponding solution accuracy for a range of solution rank values.

The numerical experiments were performed on an Intel® E5-2650 v2 CPU with 8 threads and 64 Gb RAM. We employ an Nvidia® P100 GPU card for some subtasks: The Galerkin update relies on dense linear algebra to solve (10) by the CG method, so this stage admits an efficient GPU implementation. Algorithm 1 is implemented in MATLAB® R2017b, and was run on the Balena High Performance Computing Service at the University of Bath. See Sect. 5 for additional data and code resources.

We measure errors in the solution using the root mean squared error. Given any reference solution $W_{\star }$ of size ${n_{\text{Y}}}\times {n_{\text{X}}}$, e.g. the truth or a large-rank solution when the truth is unknown, and a low-rank solution $W_{r}$, the RMS error is computed as $\mathcal{E}(W_{r},W_{\star }) = \frac{ \Vert W_{r}- W_{\star } \Vert _{F} }{\sqrt{{n_{\text{Y}}}{n_{\text{X}}}}} $. We also report the relative error in the Frobenius norm $\mathcal{E}_{\mathrm{rel}}(W _{r},W_{\star }) = \frac{ \Vert W_{r}- W_{\star } \Vert _{F} }{ \Vert W_{\star } \Vert _{F}} $.

### Test problem: a toy brain

We use the same test problem as in Harris et al. [19], a one-dimensional “toy brain.” The source and target space are $S = T = [0,1]$. The true connectivity kernel corresponds to a Gaussian profile about the diagonal plus an off-diagonal bump:

11 $$ W_{\mathrm{true}} (x,y) = e^{- (\frac{x-y}{0.4} )^{2}} + 0.9 e^{ -\frac{ (x-0.8 )^{2} + (y-0.1)^{2}}{(0.2)^{2}} } \;. $$

The input and output spaces were discretized using ${n_{\text{X}}}= {n_{\text{Y}}}= 200$ uniform lattice points. Injections are delivered at ${n_{\text{inj}}}= 5$ locations in *S*, with a width of $0.12 + 0.1 \epsilon $, where $\epsilon \sim \mathrm{Uniform}(0,1)$. The values of *X* are set to 1 within the injection region and 0 elsewhere, $\varOmega _{ij} = 1 - X_{ij}$, *Y* is set to 0 within the injection region, and we add Gaussian noise with standard deviation $\sigma = 0.1$. The matrices $L_{x} = L_{y}$ are the 3-point graph Laplacians for the 1-D chain.

**Figure 2:**
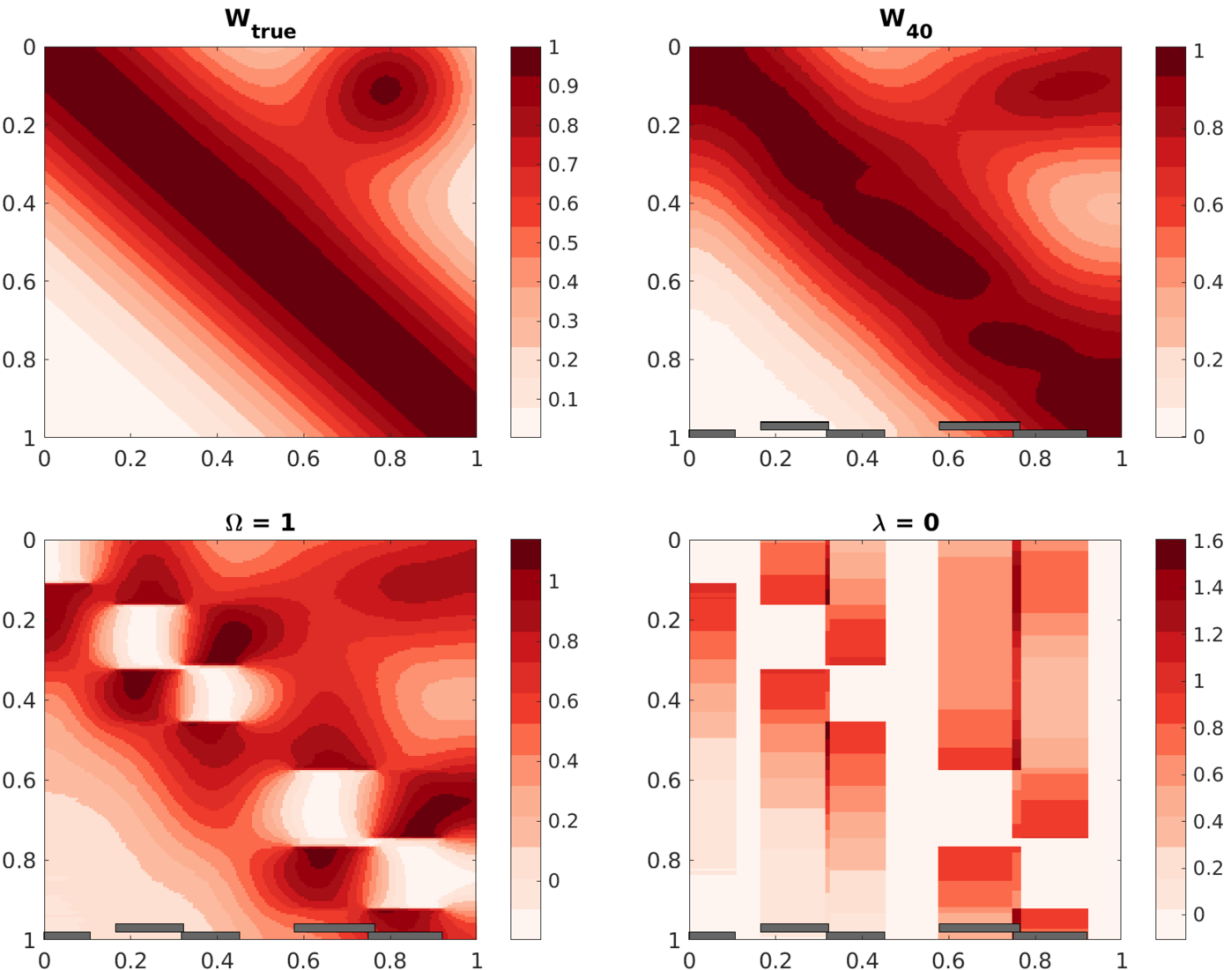
Toy brain test problem. Top: true connectivity map $W_{\mathrm{true}}$ (left) and the low-rank solution with $\mathrm{rank}=40$ and $\lambda =100$ (right). Bottom: solutions with $\varOmega =1$ (left) and $\lambda =0$ (right). The locations of simulated injections are shown by the gray bars. This shows that both the mask (*Ω*) and the smoothing ($\lambda > 0$) are necessary for good recovery

We depict the true toy connectivity $W_{\text{true}}$ as well as a number of low-rank solutions output by our method in Fig. 2. Both the mask and the regularization are required for good performance: If we remove the mask, setting *Ω* equal to the matrix of all ones, then there are holes in the data at the location of the injections. If we try fitting with $\lambda = 0$, i.e. no smoothing, then the method cannot fill in holes or extrapolate outside the injection sites. It is only with the combination of all ingredients that we recover the true connectivity.

In Table 1 we show the performance of the algorithm for ranks $r = 10$, 20, 40, 60, and 80. The output *W* is compared to $W_{\text{true}}$ as well as the rank 140 output of the algorithm. The stopping tolerance was $\tau = 10^{-7}$ to ensure that the algorithm has reached this maximal rank. We see that the RMS distance to the reference solution $W_{140}$ decreases as we increase the rank, as does the relative distance. However, the RMS and relative distances from $W_{\text{true}}$ asymptote to roughly 0.07 and 10%, respectively, by rank 40. This shows that rank 40 is a suitable maximum rank for this problem given the input data and noise.

**Table 1:** Computing times and errors for the toy brain test problem. The output *W* is compared to truth and a rank 140 reference solution

rank(*W*)	10	20	40	60	80
CPU time (sec.)	0.0396	0.1554	0.9653	2.6398	3.1108
$\mathcal{E}(W,W_{140})$	3.2324e−01	5.5407e−02	1.4162e−02	1.2125e−03	3.1549e−04
$\mathcal{E}(W,W_{\mathrm{true}})$	2.9418e−01	7.9921e−02	7.1537e−02	6.9777e−02	6.9821e−02
$\mathcal{E}_{\mathrm{rel}}(W, W_{140})$	4.3320e−01	8.9700e−02	2.4900e−02	2.5000e−03	5.1300e−04
$\mathcal{E}_{\mathrm{rel}}(W,W_{\mathrm{true}})$	4.0130e−01	1.1410e−01	1.0350e−01	1.0040e−01	1.0040e−01

The computing time of the greedy method (in this example we use the CPU only version) remains in the order of seconds even for the largest considered ranks. In contrast, the unpreconditioned CG method needs thousands of iterations (and hundreds of seconds of time) to compute a solution within the same order of accuracy. Since it is unclear how to develop a preconditioner for Eq. (5), especially for a non-trivial *Ω*, in the next sections we focus only on the greedy algorithm.

### Mouse cortex: top view connectivity

We next benchmark Algorithm 1 on mouse cortical data projected into a top–down view. See Sect. 5 for details about how we obtained these data. Here, the problem sizes are ${n_{\text{Y}}}= 44\mbox{,}478$ and ${n_{\text{X}}}= 22\mbox{,}377$ and the number of injections ${n_{\text{inj}}}=126$. We use the smoothing parameter $\bar{\lambda }=10^{6}$.

We run the low-rank solver with the target solution rank varying from $r= 125$ to 1000. The stopping tolerances *τ* were decreased geometrically from 10^−3^ for $r=125$ to 10^−6^ for $r=1000$. This delivers accurate but cheap solutions to the Galerkin system (10) while ensuring that the algorithm reached the target rank.

These low-rank solutions are compared to the full-rank solution $W_{\text{full}}$ with $r= {n_{\text{X}}}= 22\mbox{,}377$ found by L-BFGS (Byrd et al. [8]), similar to Harris et al. [19], which used L-BFGS-B to deal with the nonnegativity constraint. Note that this full-rank algorithm was initialized from the output of the low-rank algorithm. This led to a significant speedup: The full-rank method, initialized naively, had not reached a similar value of the cost function (4) after a *week* of computation, but this “warm start” allowed it to finish within hours.

**Figure 3:**
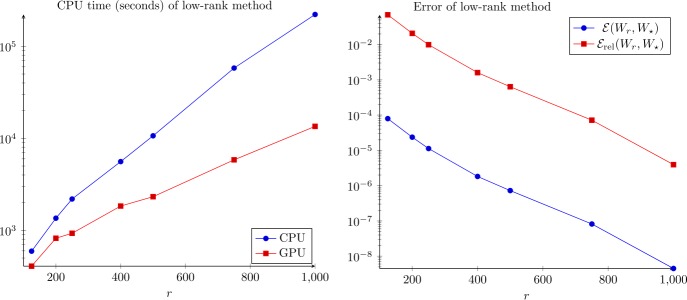
Computing times and errors for the top view data. The errors are computed with reference to the full-rank solution $W_{\star }= W_{\mathrm{full}}$. Full rank time: $\gg 6 \times 10^{5}$ s (see text)

**Figure 4:**
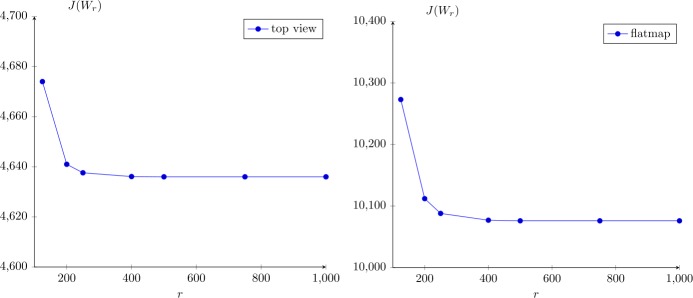
Value of cost function $J(W_{r})$ versus the rank *r* of the low-rank approximation $W_{r}$

The computing times and errors are presented in Fig. 3. We see that the RMS errors are relatively small for ranks above 500, below 10^−6^. Neither the RMS or relative error seem to have plateaued at rank 1000, but they are small. At rank 1000, the vector $\ell _{\infty }$ error (maximum absolute deviation of the matrices as vectors, not the matrix ∞-norm) $\| W_{1000} - W_{\text{full}} \|_{\infty }$ is less than 10^−6^, which is certainly within experimental uncertainty. In Fig. 4, the value of the cost function $J(W_{r})$ is plotted against the rank *r* of the approximation $W_{r}$ for the top view (left) and flatmap data (right). Apparently, around $r=500$ the cost function begins to stagnate indicating that the approximation quality does not significantly improve any more. Hence, we continue the investigation with the numerical rank set to $r=500$.

We analyze the leading singular vectors of the solution. The output of the algorithm is $W_{r} = U Z V^{\intercal }$, which is *not* the SVD of $W_{r}$ because *Z* is not diagonal. We perform a final SVD of the Galerkin matrix, $Z = \tilde{U} \varSigma \tilde{V}^{\intercal }$ and set $\hat{U} = U \tilde{U}$ and $\hat{V} = V \tilde{V}$, so that $W_{r} = \hat{U} \varSigma \hat{V}^{\intercal }$ is the SVD of the solution.

**Figure 5:**
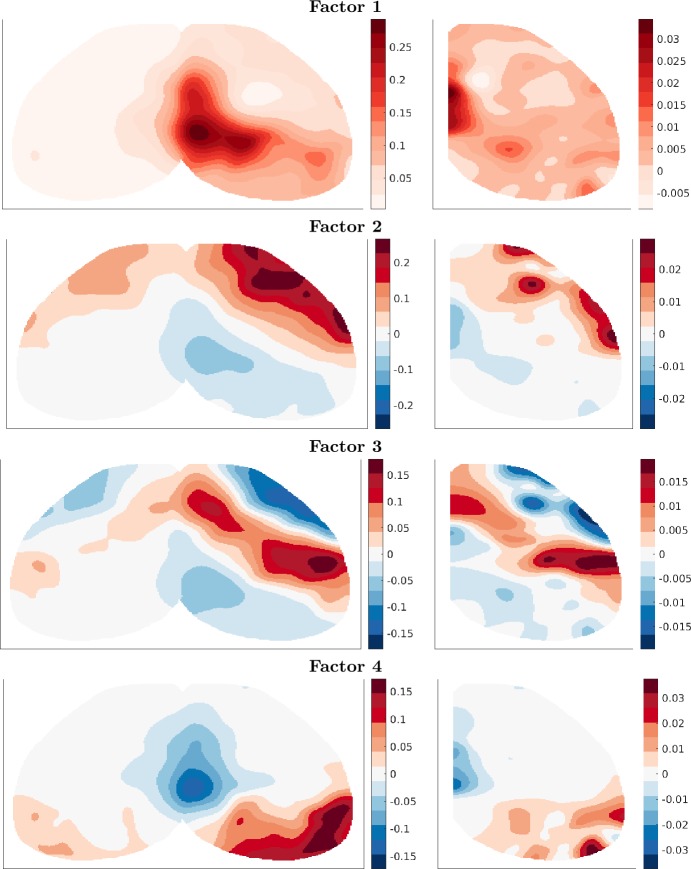
Top four singular vectors of the top view connectivity with $r= 500$. Left: scaled target vectors *ÛΣ*. Right: source vectors *V̂*

The first four of these singular vectors are shown in Fig. 5. The brain is oriented with the medial-lateral axis aligned left–right and anterior–posterior axis aligned top–bottom, as in a transverse slice. The midline of the cortex is in the center of the target plots, whereas it is on the left edge of the source plots. We observe that the leading component is a strong projection from medial areas of the cortex near the midline to nearby locations. The second component provides a correction which adds local connectivity among posterior areas and anterior areas. Note that increased anterior connectivity arises from negative entries in both source and target vectors. The sign change along the roughly anterior–posterior axis manifests as a reduction in connectivity from anterior to posterior regions as well as from posterior to anterior regions. The third component is a strong local connectivity among somatomotor areas located medially along the anterior–posterior axis and stronger on the lateral side where the barrel fields, important sensory areas for whisking, are located. Finally, the fourth component is concentrated in posterior locations, mostly corresponding to the visual areas, as well as more anterior and medial locations in the retrosplenial cortex (thought to be a memory and association area). The visual and retrosplenial parts of the component show opposite signs, reflecting stronger local connectivity within these regions than distal connectivity between them.

These patterns in Fig. 5 are reasonable, since connectivity in the brain is dominantly local with some specific long-range projections. We also observe that the projection patterns (left components *ÛΣ*) are fairly symmetric across the midline. This is also expected due to the mirroring of major brain areas in both hemispheres, despite the evidence for some lateralization, especially in humans. The more specific projections between brain regions will show up in later, higher frequency components. However, it becomes increasingly difficult to interpret lower energy components as specific pathways, since these combine in complicated ways.

### Mouse cortex: flatmap connectivity

Finally, we test the method on another problem which is a flatmap projection of the brain (see Sect. 5 for details). This projection more faithfully represents areas of the cortex which are missing from the top view since they curl underneath that vantage point. The flatmap is closer to the kind of transformation used by cartographers to flatten the globe, whereas the top view is like a satellite image taken far from the globe.

The problem size is now larger by roughly a factor of three relative to the top view. Here, ${n_{\text{Y}}}= 126\mbox{,}847$ and ${n_{\text{X}}}= 63\mbox{,}435$. The number of experiments is the same, ${n_{\text{inj}}}=126$, whereas the regularization parameter is set to $\bar{\lambda }=3 \times 10^{7}$. The smoothing parameter was set to give the same level of smoothness, measured “by eye,” in the components as in the top view experiment. The tolerances *τ* were as in the top view case.

**Figure 6:**
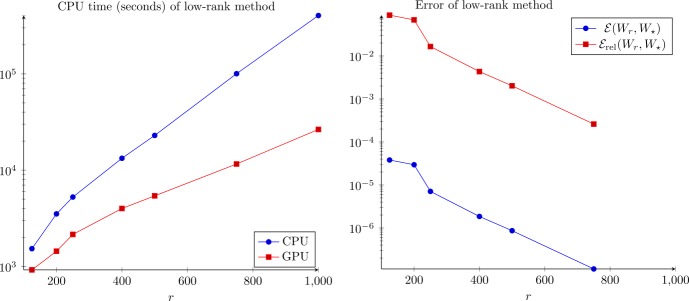
Computing times and errors for the flatmap data. The errors are computed with reference to the rank-1000 solution $W_{\star }= W_{1000}$

In this case, the computing time of the full solver would be excessively large, so we do not estimate the error by comparison to the full solution, instead taking the solution with $r= 1000$ as the reference solution $W_{\star }= W_{1000}$. The computing times and the errors are shown in Fig. 6. Here, the benefits by using the GPU implementation for solving (10) were more significant than for the top view case. We obtained the rank 500 solution in approximately 1.5 hours, which is significantly less than with the pure CPU implementation, which took 6.4 hours. Comparing Figs. 3 and 6, the computation times for the flatmap problem with $r= 500$ and 1000 are roughly twice as large as for the top view problem. On the other hand, for $r= 125$ and 250, the compute times are about three times as long for flatmap versus top view. The observed scaling in compute time appears to be slightly slower than $\mathcal{O}(n)$ in these tests. The growth rate of the computing time on the GPU is better than that of the CPU version since the matrix multiplications, which dominate the CPU cost for large ranks, are calculated in nearly constant time, mainly due to communication overhead, on the GPU. The RMS error between rank 500 and 1000 is again less than 10^−6^, so we believe rank 500 is probably a very good approximation to the full solution. Figure 4 (right) shows the costs versus the approximation rank. Again, we see that $r=500$ is reasonable and the distance from $W_{\star}$ is smaller than 10%.

**Figure 7:**
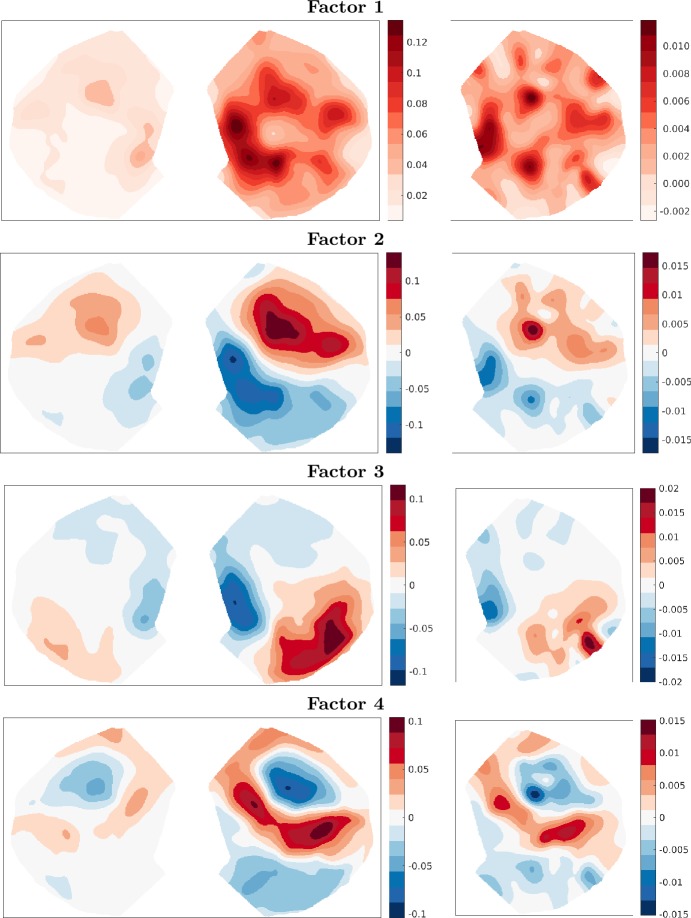
Top four singular vectors of the flatmap connectivity with $r= 500$. Left: scaled target vectors *ÛΣ*. Right: source vectors *V̂*

The four dominant singular vectors of the flatmap solution are shown in Fig. 7, oriented as in Fig. 5, with the anterior–posterior axis from top–bottom and the medial-lateral axis from left–right. The first two factors are directly comparable between the two problem outputs, although we see more structure in the flatmap components. This could be due to employing a projection which more accurately represents these 3-D data in 2-D, or due to the choice of smoothing parameter *λ̄*. The third and fourth components, on the other hand, are comparable to the fourth and third components in the top view problem, respectively. Again, these patterns are reasonable and expected. The raw 3-D data that were fed into the top view and flatmap projections were the same, but the greedy algorithm is run using different projected datasets. It is reassuring that we can interpret the first few factors and directly compare them against those in the top view.

### Dropping the nonnegativity constraint does not strongly affect the solutions

In order to apply linear methods, we relaxed the nonnegativity constraint when formulating the unconstrained problem (4), as opposed to the original problem with nonnegativity constraint (1). We now show that the resulting solutions are not significantly different between the two problems. This justifies the major simplification that we have made.

In all of our experiments with the test problem (Sect. 3.1), the resulting matrices were nearly nonnegative. The solution $W_{40}$ has 48 out of 40,000 negative entries. These negative entries were all greater than −0.0023 in the lower-left corner of the matrix (see Fig. 2), where the truth is approximately zero.

We were able to solve the top view problem with the nonnegative constraint using L-BFGS-B by initializing with $W_{\mathrm{full}}$ projected onto the nonnegative orthant. Let $W_{\mathrm{proj}}$ be the matrix with entries $(W_{\mathrm{proj}})_{ij} = \max (0, (W_{\mathrm{full}})_{ij})$, and let $W_{\mathrm{nonneg}}$ denote the solution to the constrained problem obtained in this way. Comparing the nonnegative versus unconstrained solutions, we found that $\mathcal{E}(W_{\mathrm{full}}, W_{\mathrm{nonneg}}) = 3.99\mbox{e}{-}04$. Projecting $W_{\mathrm{full}}$ onto the nonnegative orthant leads to $\mathcal{E}(W_{\mathrm{proj}}, W_{\mathrm{nonneg}}) = 3.67\mbox{e}{-}04$. In either case the ∞-norm difference is 0.009. These results show that the solution to the unconstrained problem is close to the solution of the constrained problem, and that the projection of the solution to the unconstrained problem is also close to the constrained solution. Algorithm 1 thus offers an efficient way to approximate the solution to the more difficult nonnegative problem, while retaining low rank.

## Discussion

We have studied a numerical method specifically tailored for the important neuroscience problem of connectome regression from mesoscopic tract tracing experiments. This connectome inference problem was formulated as the regression problem (4). The optimality conditions for this problem turn out to be a linear matrix equation in the unknown connectivity *W*, which we propose to solve with Algorithm 1. Our numerical results show that the low-rank greedy algorithm, as proposed by Kressner and Sirković [26], is a viable choice for acquiring low-rank factors of *W* with a computation cost that was significantly smaller compared to other approaches (Harris et al. [19]; Benner and Breiten [3]; Kressner and Tobler [27]). This allows us to infer the flatmap matrix, with approximately 140× more entries than previously obtained for the visual system, while taking significantly less time: computing the flatmap solution took hours versus days for the smaller low-rank visual network (Harris et al. [19]). The first few singular vector components of these cortical connectivities are interpretable and reasonable from a neuroanatomy standpoint, although a full anatomical study of this inferred connectivity is outside the scope of the current paper.

The main ingredients of Algorithm 1 are solving the large, sparse linear systems of equations at each ALS iteration and solving the dense but small projected version of the original linear matrix equation for the Galerkin step. We had to carefully choose the solvers for each of these phases of the algorithm. The Galerkin step forms the principal bottleneck due to the absence of direct numerical methods to handle dense linear matrix equations of moderate size. We employed a matrix-valued CG iteration to approximately solve (10), implementing it on the GPU for speed. This lead to cubic complexity in *r* at this step. One could argue that equipping this CG iteration with a preconditioner could speed up its convergence, but so far we were not successful in finding a preconditioner that both reduced the number of CG steps and the computational time. A future research direction could be to derive an adequate preconditioning strategy for the problem structure in (7), which would increase the efficiency of any Krylov method.

Matrix-valued Krylov subspace methods (Damm [13]; Kressner and Tobler [27]; Benner and Breiten [3]; Palitta and Kürschner [36]) offer an alternative class of possible algorithms to solving the overall linear matrix equation (7). However, for rapid convergence of these methods we typically need a preconditioner. In our tests on (7), these approaches performed poorly, because rank truncations (e.g. via thin QR or SVD) are required after major subcalculations which occur at every iteration. Computing these decompositions quickly became expensive because of the sheer amount of necessary rank truncations in the Krylov method. If a suitable preconditioner for our problem would be found, it would make sense to give low-rank matrix-valued Krylov methods another try.

The original regression problem proposed by Harris et al. [19] (1) demands that the solution *W* be nonnegative. So far, this constraint is not considered by the employed algorithm. However, for the test problem and data we have tried, the computed matrix turns out to be majority nonnegative. We find typically small negative entries that can be safely neglected without sacrificing accuracy. Although a mostly nonnegative solution is not generally expected when solving the unconstrained problem (4), it appears that such behavior is typical for nonnegative data matrices *X* and *Y*.

Working directly with nonnegative factors $U \geq 0$ and $V \geq 0$ was originally proposed by Harris et al. [19], where they applied a projected gradient method to find such an approximation for connectome of mouse visual areas albeit very slowly. Such a formulation is preferred, since it leads to a nonnegative *W*, and it allows interpreting the leading factors as the most important neural pathways in the brain. Modifying Algorithm 1 to compute nonnegative low-rank factors or enforcing that the low-rank approximation $UV^{\intercal }\approx W$ is nonnegative—a nonlinear constraint—is a much harder goal to achieve. For instance, even if one generated nonnegative factor matrices *U* and *V*, e.g. by changing the ALS step to nonnegative ALS, the orthogonalization and Galerkin update each destroy this nonnegativity. New methods of NMF which incorporate regularizations similar to our Laplacian terms (Cichocki et al. [12]; Cai et al. [9]) are an area of ongoing research, and the optimization techniques developed there could accelerate the nonnegative low-rank formulation of (1). These include other techniques developed with neuroscience in mind, such as neuron segmentation and calcium deconvolution (Pnevmatikakis et al. [38]) as well as sequence identification (Mackevicius et al. [29]). The greedy method we have presented is an excellent way to initialize the nonnegative version of the problem, similar to how SVD is used to initialize NMF. We hope to improve upon nonnegative low-rank methods in the future.

Model (1) is certainly not the only approach to solving the connectome inference problem. The loss term $\| P_{\varOmega }(WX - Y) \|_{F}^{2}$ is standard and arises from Gaussian noise assumptions combined with missing data and is standard loss in matrix completion problems with noisy observations (e.g. Mazumder et al. [31]; Candes and Plan [10]). The regularization term is a thin plate spline penalty (Wahba [48]). This is one of many possible choices for smoothing, among them penalties such as $\| \mathrm{grad}(W) \|^{2}$ or the total variation semi-norm (Rudin et al. [42]; Chambolle and Pock [11]), which favors piecewise-constant solutions. While we recognize that there are many possible choices for the regularizer, the thin plate penalty is reasonable, linear and thus convenient to work with. Previous work (Harris et al. [19]) has shown that it is useful for the connectome problem. Testing other forms of regularization is a worthy goal but not straightforward to implement at scale. This is outside the scope of the current paper.

Finally, the most exciting prospects for this class of algorithms is what can be learned when we apply them to next-generation tract tracing datasets. Such techniques can be used to resolve differences between the rat (Bota et al. [6]) brain and mouse (Oh et al. [34]), or uncover unknown topographies (see Reimann et al. [40]) in these and other animals (like the marmoset, Majka et al. [30]). The mesoscale is also naturally the same resolution as obtained by wide-field calcium imaging. Spatial connectome modeling could elucidate the largely mysterious interactions different sensory modalities, proprioception, and motor areas, hopefully leading to better understanding of integrative functions.

## Data and code

We tested our algorithm on two datasets (top view and flatmap) generated from Allen Institute for Brain Science Mouse Connectivity Atlas data http://connectivity.brain-map.org. These data were obtained with the Python SDK allensdk version 0.13.1 available from http://alleninstitute.github.io/AllenSDK/. Our data pulling and processing scripts are available from https://github.com/kharris/allen-voxel-network.

We used the allensdk to retrieve 10 *μ*m injection and projection density volumetric data for 126 *wildtype* experiments in cortex. These data were projected from 3-D to 2-D using either the top view or flatmap paths and saved as 2-D arrays. Next, the projected coordinates were split into left and right hemispheres. Since *wildtype* injections were always delivered into the right hemisphere, this becomes our source space *S* whereas the union of left and right are the target space *T*. We constructed 2-D 5-point Laplacian matrices on these grids with “free” Neumann boundary conditions on the cortical edge. Finally, the 2-D projected data were downsampled 4 times along each dimension to obtain 40 *μ*m resolution. The injection and projection data were then stacked into the matrices *X* and *Y*, respectively. The mask *Ω* was set via $\varOmega _{ij} = 1_{\{ X _{ij} \leq 0.4 \}}$.

MATLAB code which implements our greedy low-rank algorithm (1) is included in the repository: https://gitlab.mpi-magdeburg.mpg.de/kuerschner/lowrank_connectome. We also include the problem inputs *X*, *Y*, $L_{x}$, $L_{y}$, *Ω* for our three example problems (test, top view, and flatmap) as MATLAB files. Note that *Ω* is stored as $1-\varOmega $ in these files, as this matches the convention of (Harris et al. [19]).

## Footnotes

1 In this paper, we take a different convention for *Ω* (the complement) as in Harris et al. [19].

2 KD Harris, personal communication, 2017. Note that these times are for the much smaller visual areas dataset.

3 Wildtype injections can cover 30–500 voxels, approximately 240 on average, at 100 *μ*m resolution (Oh et al. [34]).

4 One may consider rescaling *λ* as before, but subtle differences arise. In the continuous versus discrete cases the units of the equations are different, since the functions $X_{i}(x)$ and $Y_{i}(y)$ are now viewed as densities. Furthermore, there is a mismatch in units between (1) and (2), because the graph Laplacian is unitless whereas the Laplace operator is not. This explains the lack of any dependence on the grid size in the scaling of the discrete problem. Regardless, choosing the exact scaling to make the continuous and discrete cases match is not necessary for the more qualitative argument we are making.

## Abbreviations

MOp: primary motor cortex
L-BFGS: Limited-memory Broyden–Fletcher–Goldfarb–Shanno algorithm
L-BFGS-B: L-BFGS with Box constraints
*d*-D: *d*-dimensional
ALS: Alternating Linear Scheme
Eq.: Equation
CG: Conjugate Gradient (method)
CP: Canonical Polyadic (decomposition)
GPU: Graphics Processing Unit
RMS: Root Mean Square (error)
SVD: Singular Value Decomposition
NMF: Nonnegative Matrix Factorization
